# Understandings of reproductive tract infections in a peri-urban pueblo joven in Lima, Peru

**DOI:** 10.1186/1472-6874-6-7

**Published:** 2006-05-02

**Authors:** Lisa Scipioni Hernández, Peter J Winch, Kea Parker, Robert H Gilman

**Affiliations:** 1 Independent Consultant, New York, New York, USA; 2 Department of International Health, Johns Hopkins Bloomberg School of Public Health, Baltimore, Maryland, USA; 3 Medical Student, Leonard M. Miller School of Medicine at the University of Miami, Miami, Florida, USA

## Abstract

**Background:**

Control programs for Reproductive Tract Infections (RTIs) typically focus on increasing awareness of risks associated with different forms of sexual contact, and pay little attention to how or why people may link RTIs to other features of their physical or social environments. This paper describes how women in a peri-urban pueblo joven located in the coastal desert surrounding Lima, Peru conceptualize the links between RTIs, sexual behaviour, personal hygiene, and the adverse environment in which they live.

**Methods:**

We combined qualitative interviews and a participatory voting exercise to examine social and physical environmental influences on RTIs and gynaecologic symptom interpretation.

**Results:**

Knowledge of RTIs in general was limited, although knowledge of AIDS was higher. Perceived causes of RTIs fell into three categories: sexual contact with infected persons, personal hygiene and exposure to the contaminated physical environment, with AIDS clearly related to sexual contact. The adverse environment is thought to be a major contributor to vaginal discharge, "inflamed ovaries" and urinary tract infection. The more remote parts of this periurban squatter settlement, characterized by blowing sand and dust and limited access to clean water, are thought to exhibit higher rates of RTIs as a direct result of the adverse environment found there. Stigma associated with RTIs often keeps women from seeking care or obtaining information about gynaecologic symptoms, and favours explanations that avoid mention of sexual practices.

**Conclusion:**

The discrepancy between demonstrated disease risk factors and personal explanations influenced by local environmental conditions and RTI-related stigma poses a challenge for prevention programs. Effective interventions need to take local understandings of RTIs into account as they engage in dialogue with communities about prevention and treatment of RTIs.

## Background

Reproductive Tract Infections (RTIs) are a major cause of ill health globally [1,2]. RTIs can be caused by sexually transmitted infections (STIs), overgrowth of organisms normally present in the reproductive tract, and medical and surgical procedures including insertion of intrauterine devices and induced abortions. In women, RTIs can be asymptomatic, and even when symptomatic their presentation can overlap with and be misdiagnosed as normal physiologic change, and normal physiologic discharge may be misdiagnosed as RTI [3]. RTIs are extremely common in Peru. A recent study on RTIs conducted on a sample drawn from the coastal, highland and jungle regions of Peru found that 77% of women reported symptoms compatible with RTIs, 70.4% were found positive for at least one RTI and 38.4% were positive for two or more RTIs [4]. Bacterial vaginosis was found in 43.7% of the sample, trichomoniasis in 16.5%, vulvovaginal candidiasis in 4.5%, Chlamydia in 6.8%, gonorrhoea in 1.2% and cervical human papilloma virus infection in 4.9% [4]. Another recent study on herpes simplex virus type 2 (HSV-2) found a 20.5% prevalence in women and 7.1% in men in the general population in coastal Peru [5].

This paper focuses on local understandings of the causation (aetiology) of RTIs in peri-urban Lima, Peru, and ways in which women link the occurrence of RTIs to environmental and living conditions in the communities where they live. Local models of illness causation are only one aspect of a larger pattern of cultural response to RTIs, but they have been a specific focus of qualitative studies on RTIs for several reasons. First, rates of careseeking for RTIs from allopathic (cosmopolitan medical) providers are generally reported to be low. There is an assumption, often not clearly articulated in research reports, that if women ascribe RTIs to infectious agents they will be more likely to utilize modern medical care including antibiotics, whereas if they ascribe RTIs to other causes such as lack of personal hygiene, adverse environmental conditions, dietary imbalance or supernatural forces, they will try other treatments such as traditional remedies. A second assumption is that, if it is not understood that sexual contact is an important cause of RTIs, this may favour the adoption of prevention methods unrelated to safer sexual practices such as changes in diet or wearing of amulets.

Table 1 summarizes the perceived causes of RTIs including sexual transmission reported in six qualitative studies [6-11] and one quantitative study from Brazil [12]. This presentation is illustrative rather than comprehensive, and were selected from approximately 35 qualitative studies on RTIs identified through a search on PubMed because 1) they provided significant detail (at least three paragraphs) on local understandings of RTI causation and 2) they represent a range of settings. In the interest of brevity, not every possible cause reported in these 7 articles is included in Table 1. Although it is difficult to discern an overall pattern to the perceived causes cited in Table 1 and other articles, RTIs are primarily attributed to contamination with germs or unclean substances through various routes such as sexual contact, ingestion of contaminated food, use of unclean needles, or an internal imbalance or over-exertion. The action of malevolent agents is significant in some settings, leading people to take measures to protect themselves from spirits [7], or consult traditional practitioners who specialize in illnesses related to witchcraft and sorcery [10,11,13]. The concept of contamination may include the transfer of infectious agents occurring through sexual and other forms of contact, but also includes concerns about exposure to dirt. Linking of the concept of contamination to notions of purity and pollution are most prevalent in South Asia [6,7,14].

**Table 1:** Perceived causes of reproductive tract infections among informants reported in seven primarily qualitative studies

Causes of RTIs mentioned by study participants		Pakistan	Bangla-desh	Vietnam	Liberia	South Africa	Uganda	Brazil
		
		[6]	[7]	[8]	[9]	[10]	[11]	[12]
**1. CONTAMINATION WITH GERMS OR UNCLEAN SUBSTANCES THROUGH VARIOUS ROUTES**								
1.1 Sexual contact with infected persons		No	Yes	Yes	Yes	Yes	Yes	Yes
1.2 Personal hygiene	Hygiene before and after sexual contact	No	No	Yes	No	Yes	No	No
	Infrequent washing of genitals, unclean undergarments or towels	Yes	No	Yes	Yes	Yes	Yes	Yes
	Poor hygiene during or after menstruation	No	No	Yes	No	No	No	No
	Sharing toilets, dirty toilets	No	No	No	Yes	No	Yes	Yes
1.3 Exposure to contaminated physical environment	Exposure to contaminated water	Yes	No	Yes	No	No	No	No
	Exposure to contaminated soil, dirt or dust carried by air	Yes	No	No	Yes	No	No	No
	Stepping in infected urine	No	No	No	Yes	No	No	No
1.4 Pubic lice, other biting insects		No	No	No	No	Yes	Yes	No
1.5 Surgical procedures: IUD insertion, tubal ligation, induced abortions		Yes	Yes	Yes	No	No	No	No
1.6 Intravenous drug use		No	No	No	No	No	No	Yes
**2. INTERNAL IMBALANCE**								
2.1 Diet, dietary imbalance		Yes	No	Yes	Yes	No	No	No
2.2 Weakness or excessive demands on body	Hard work	No	Yes	No	No	No	Yes	No
	Weakness	Yes	No	No	No	No	No	No
	Pregnancy and childbirth	Yes	Yes	No	No	No	No	No
**3. ACTION OF EXTERNAL AGENTS**								
3.1 Malevolent spirits		No	Yes	No	Yes	No	No	No
3.2 Will of God, Nature's will		Yes	Yes	No	No	No	No	No
3.3 Witchcraft and sorcery		No	No	No	No	Yes	Yes	No

While sexual transmission is often the most commonly mentioned cause, in one study in Karachi, Pakistan, none of the women interviewed nor the health care providers they had consulted had suggested sexual transmission as a possible cause of RTIs [6]. Results from Vietnam are typical of many studies, where "almost every woman knows that some infections can be sexually transmitted, but they consistently report that it is not the case for their own symptoms" [8]. Stigma associated with sexual transmission of diseases commonly appears to lead both men and women to favour other causal explanations.

Personal hygiene stands at the intersection of many causal explanations, and is a common way informants attempt to lessen the risks of sexual relations they may not be in a position to avoid. Personal hygiene may include washing before and after sexual relations, wearing clean undergarments, avoiding contact with toilet seats and using disinfectants. In Kenya, Nzioka reports that "the act of washing or taking a shower which lead to physical cleanliness" is a form of safer sex [15]. A study in North India found nearly half the young men in a survey believed that washing the penis with a disinfectant after sex helped prevent disease, and 30–40% continued to believe that urinating after sex reduced the chances of transmission of sexually-transmitted infections, even at the conclusion of an intensive community-based education intervention [16].

Personal hygiene is also thought to be the vehicle through which the physical environment (exposure to dirt, dust, dirty water) increases the likelihood of RTIs. When the physical environment is mentioned as having a role in RTIs, personal hygiene is usually mentioned as a factor mediating that effect. In Vietnam, working waist deep in water is seen as a prime cause of RTIs, and has two aspects: exposure to infection through the dirty water itself, and the dampness itself, spending all day in damp clothing [8]. Women reported douching with a solution containing boiled guava leaves and salt to "dry out the vagina" [8].

In this paper we describe the links women in a peri-urban neighbourhood near Lima, Peru make between RTIs and the physical environment in which they live, and how they perceive that the risk of RTIs varies according to where one lives. These data will demonstrate that managers of programs seeking to reduce the impact of RTIs need to take into consideration not only the social environment in which people live, including gender relations, but also their physical environment and neighbourhood characteristics.

## Methods

### Study site

Research was conducted in Las Pampas de San Juan de Miraflores, a peri-urban pueblo joven (young town), located 15 kilometres south of urban Lima with a total population of 40,700. Physically, this environment consists of steep and rocky desert hills with dusty soil, no natural vegetation, and less than one inch of rainfall a year. A low layer of clouds, created when cold Pacific winds meet the Andes mountain range to the east of Lima, covers the city for 9 months of the year [17].

Pueblos jovenes are nearly always formed by well-planned community efforts, often in the form of illegal "land invasions" [18]. Literally overnight, prospective residents occupy an undeveloped tract of land and each family quickly constructs a home using whatever material is available. As communities grow, economic distinctions become visible; more established residents live in lower elevations, have homes built with cement blocks and have better access to resources and transportation. We will refer to these areas as "modern" Pampas. Homes located at higher elevation are built with woven bamboo mats, plastic, and other scavenged material. These areas are occupied by more recent arrivals to the community and we will refer to them as "marginal" Pampas. Inhabitants of pueblos jovenes work together to transform newly formed settlements into communities equipped with amenities such as water, electricity, schools, medical centres, and police stations [17].

Las Pampas, like other pueblos jovenes, is characterized by high rates of underemployment and poverty. In 2000, the annual reported median income for heads of households in this community was $2100 and income ranged from $850 to $4600 per year [19]. Most households have electricity but only about half have water or sewage connections. For those without water connections, water must be purchased either from neighbours with connections, from private water sources, or from pilones, water connections whose service and expense are shared by neighbours. In part because of this, water use is extremely low. Families in Las Pampas on average use only 75 litres of water a day and less than 15% of total water is used for bathing [20]. Bathroom facilities for residents without indoor plumbing are called silos (outhouses) and are often shared by numerous families.

### Semi-structured interviews

During the 7 weeks between October 21 and December 10, 1997 the first author was located at a health post serving Las Pampas de San Juan de Miraflores with a female paramedic specialized in women's health or *obstetriz*. Women who came to the health post to visit the obstetriz specifically were asked if they would be willing to participate in the study. The obstetriz and researcher were present in the health post from 10-3 Monday-Thursday. The health post was staffed by a physician Mondays-Thursdays during the same time. All women who attended the health post were invited to participate in semi-structured interviews. Interviews were conducted in a private room in the health post. Approximately 200 women were approached to participate, and 32 agreed to participate in the study. Of these, 3 agreed to and completed an additional interview. Interviews lasted between 1 and 2 hours. Topics covered included: community health concerns; RTI awareness, prevention, causes, and available treatment; and social factors affecting sexuality and health seeking behaviour. Interviews and activities took place in Spanish and were carried out by the same female researcher (L.S.H.). Those who declined to participate mentioned time constraints and apprehension in discussing the subject matter as reasons for not participating. Ages ranged from 17 to 45 years; 69% of respondents were between 20 and 34 years old and the mean age was 28. All participants were self-reported mestizo (of mixed indigenous and Spanish background). One of the women reported no formal education and three had completed university training. The majority (68%) had some secondary education but had not finished high school. Most (66%) of the respondents were not born in Lima. Age at first sexual relation was 8–9 (1 respondent), 10–15 (3 resp.), 16–19 (19 resp.) and 20–24 (9 resp.). The first partner was reported to be a friend (1 resp.), boyfriend (18 resp.), spouse (5 resp.), roommate (3 resp.), stranger (2 resp.), family member (1 resp.) or the first encounter was as a victim of rape (2 resp.). The reported number of lifetime partners was 1 (16 resp.), 2 (3 resp.), 3 (10resp.) and more than 3 (2 resp.) with a mean of 2. Three of the women reported ever having used condoms. Interviews were tape recorded with the participant's consent and then translated into English. Responses were searched, coded thematically, and analyzed using Nud*ist (Non-numerical Unstructured Data Indexing Searching and Theorizing) software (Version 4, 1997).

### Participatory exercise on illness occurrence and distribution

In a subsequent phase of the study, participatory data collection was conducted with a different subpopulation of the Las Pampas community. Women were visited in their homes and 23 were selected on the basis of their geographic distribution throughout Las Pampas and their willingness to participate. Approximately equal numbers were selected from women living in completed concrete box houses, women living in concrete houses under construction and women in marginal areas living in houses made of scrap materials. A participatory method was utilized to examine how they perceived geographic variation and risk of a range of reproductive health problems. Respondents were asked where certain diseases or symptoms associated with sexuality (vaginal discharge, "infection in the ovaries", urinary tract infection, AIDS and venereal diseases) occurred most frequently in their community and their reasons for this perception of disease occurrence. To answer this, participants were asked to distribute 8 beans between pictorial representations of four geographic areas according to what they perceived to be the relative frequency of occurrence of different health problems in each location. It was felt that these four geographic areas represented four distinct environments that are familiar to all residents of Las Pampas and frequently alluded to in interviews. The four locations used for this exercise were:

1. Sierra or Highlands – Rural and relatively traditional highland communities, where indigenous languages are frequently spoken, one of the main sources of migrants settling in *pueblos jovenes *(Young towns);

2. "Marginal" Pampas – Area within the *pueblo joven *of Las Pampas that is the least established, most recently developed, farthest from main roads, and highest on the surrounding hills. Structures in this area are mostly made of woven straw matting, cardboard, and corrugated iron, and rarely have electricity, sewage, or water services;

3. "Modern" Pampas – Area within the *pueblo joven *of Las Pampas that is more established than the marginal communities and is located closer to main roads. Most structures are concrete with electricity, sewage, and water connections; and

4. Urban Lima – Long-established urban environment with better access to services.

Ethical approval for this project was received from the Internal Review Board at Johns Hopkins University and A.B. PRISMA, the sponsoring Peruvian non-governmental organization with which we collaborated. We received written, informed consent from all participants and confidentiality was assured and maintained in every aspect of this study.

## Results

### Awareness of RTIs and AIDS and local terminology

The level of knowledge about RTIs in general was low. When asked about health problems in the community, only one woman mentioned RTIs or AIDS in her initial response. Another respondent, when asked if RTIs were a problem in her community, responded:

"I don't think so. I don't think so. Like I say, it may be the case that women have this disease and men have these diseases but they don't say it, they don't talk about it and well, they go and cure themselves, but they talk about this very little. I have heard little about venereal disease maybe because people don't talk about it. They don't talk."

Blame was placed on individuals for their lack of knowledge. One respondent explained:

"Ah, a lot of women don't know about these diseases. Yes, I know because I always go to the health post, with the gynaecologists, they explain it...there are a lot of women that are unaware of all this, they don't know because they don't go to the health posts or they don't prepare. There are uninformed husbands that don't explain to their wives all this; yes there are a lot of women that do not know any of these things. The health posts are there to orient us about this, but there are women that are lazy."

In contrast, another respondent stated:

"At the health post they give us very little information. Sometimes women don't go because they are embarrassed, they don't inform us enough."

Respondents stated that it was often embarrassing to go to the main health post to seek treatment for a gynaecologic infection, and that the social consequences of being perceived as having a RTI could be more detrimental than the infection itself.

Participants appeared to lack specific vocabulary to describe sexually transmitted diseases, gynaecologic symptoms, or the female genital region. Most women were familiar with the term SIDA (Spanish acronym for AIDS) and when women appeared confused by the terms *Enfermedades Transmitadas Sexualmente *(Sp. sexually transmitted disease) and *Enfermedades venereas *(Sp. venereal diseases) the researcher occasionally would resort to the explanation that they are diseases "like SIDA". The Spanish translation for sexually transmitted infection was not familiar at all to respondents. When referring to sexual transmission of disease, terms such as *contagio *(Sp. contagion), *infectado *(Sp. infected) and *quemarse *(Sp. to burn oneself) were used by the respondents. The latter is used in ways such as *lo quemaste *(Sp. you burned him) implying "you infected him with a sexually-transmitted infection". *Infecciones de los ovarios *(Sp. infections of the ovaries) is a term commonly used to describe lower abdominal pain that may be accompanied by vaginal discharge. *Descensos *(Sp. Unloading, discharge) or *enfermedades de la mujer *(Sp. illnesses of women) are terms used to describe vaginal discharge. *Abajo *(Sp. below) was used to describe the female abdominal or genital region.

### Perceived causes of RTIs: sexual contact with infected persons

Referring to the classification of perceived causes of RTIs in Table 1, the causes mentioned by study participants all fell into three categories: sexual contact with infected persons, personal hygiene and exposure to the contaminated physical environment. Biting insects, surgical procedures, drug use, diets and the actions of malevolent agents such as spirits were not mentioned, although hard work was mentioned in relation to the adverse environment.

A number of participants referred to sexual transmission as a cause of RTIs. Nevertheless, women reported that they are discouraged from appearing informed regarding sexuality. Thus even if women are aware of biomedical models of transmission, other causes related to personal hygiene or the physical environment are more acceptable as explanations for disease symptoms.

"Concerning sexuality, there are many taboos for women, they should not appear to be informed."

Health care workers are aware of the stigma associated with sexual transmission of diseases, and may avoid confronting clients about this issue. One respondent who tested positive for Chlamydia asked:

"Tell me is it sexually transmitted? Because when I went to the doctor, when my disease began recently...the doctor told me, 'don't worry, they came about because of the humidity' he didn't tell me that it was sexually transmitted."

Respondents acknowledged a link between disease spread and male infidelity. One woman who tested positive for two RTIs explained that it is important to use condoms, even though she and her partner never had:

"Because who knows if they have other women in another place that can infect us."

### Perceived role of sexual contact in AIDS transmission

Women in our study often did not clearly distinguish between AIDS and other RTIs, but they were much clearer about the relation between AIDS transmission and sexual contact than they were for other RTIs referred to by terms such as vaginal discharge or inflamed ovaries. Due in part to success of recent AIDS awareness campaigns [21], most of our respondents understood that AIDS could be transmitted sexually or through blood,

"(AIDS can be transmitted) through sex and if the blood of an infected person runs into that of a healthy person."

Our mostly monogamous informants noted multiple partners and commercial sex work as factors influencing the spread of AIDS.

"People get infected with AIDS through relations. There are men that want to have four or five women and there is one woman that has the disease and this is how they are transmitted."

"If a person stays in their house, they can't get infected with this, but those that go to the streets (for sex) can."

However, a great number of informants thought a number of additional modes of transmission existed, often through apparently casual contact.

"AIDS is transmitted through food or through a kiss."

"Through hair cuts, it could happen at the hairdressers, in the same case someone could get infected through blood"

"Another way is in bathrooms, those women go to the bathroom and they get infected"

"Through conversation"

### Perceived causes of RTIs: personal hygiene

Personal hygiene was a common explanation for the occurrence of RTIs other than AIDS. Good personal hygiene was viewed as a way of decreasing the possibility of both disease transmission from a partner, and contamination from the surrounding environment:

"Depends on the cleanliness of the woman, of the man also...(disease comes) when we don't wash."

As in Vietnam [8], dampness was a major concern. It was common for women to mention the importance of clean undergarments in preventing transmission:

"My mother said to me, it is because I use her underwear. I think that it started because of this and she told me not to use her underwear."

One respondent who tested positive for trichomoniasis and Chlamydia explained how she became infected:

"It must be after I went to the bathroom to urinate and wet my underwear and didn't change them."

When asked if she thought it could be due to sex, she responded:

"No, my partner is very careful with this."

"Careful" may refer to personal hygiene, rather than to use of condoms and avoidance of multiple partners.

"Before having a sexual relation they must both practice good hygiene"

"...when one already has sexual contact you have to clean up despite the fact that you have sex with your husband, by all means he must clean before he has sex with us."

Some women felt the cause of disease was more related to personal hygiene than multiple partners:

"Because maybe the husband goes to bed with some women that aren't clean and those women maybe are women of the streets, or also when women don't wash themselves well, I think that this is how it must be."

Problems related to personal hygiene were seen as particularly common in Las Pampas due to shortages of water, especially at higher elevations, making it difficult for women to wash regularly.

### Perceived causes of RTIs: adverse physical environment

Although both sexual contact with infected persons and lack of personal hygiene were commonly mentioned as causes of RTIs, many of our respondents attributed their own symptoms to the adverse local environment. When asked about illnesses that affect women in their community, respondents mentioned the adverse physical environment of the Las Pampas. The adverse environment is thought to produce ill health via two mechanisms. The first mechanism is contact with dirty or contaminated air, dust or soil and lack of water to maintain personal hygiene. When asked to explain why she thought vaginal discharge was a problem most common in marginal areas in Las Pampas, a recent immigrant from the provincial highland region of Peru described:

"This is a problem here mostly because of the poverty, we don't have water. In the provinces we have water in the river – here there is more dust, dirt, sand and filth."

Other women explained that symptoms come:

"From the sand, sometimes we feel it when we wear skirts"

or because:

"It is not so clean here, there are microbes in the dirt."

The second mechanism is overwork, and overexposure to cold winds, which could be classified as an imbalance according to Table 1. Both "infection in the ovaries" and vaginal discharge were commonly related to the physically gruelling lifestyle that often involves walking and carrying water and heavy loads within Las Pampas.

"(infection in the ovaries comes) from the cold, there is too much wind"

"They walk more here, it is because of the height of where they live, to go to work, they go up, and they go down these cliffs – too much travel."

Another respondent states that the combination of exposure to dirt and lack of money to purchase soap was what most aggravated the situation:

"This disease comes because there is much work and much walking...they walk through the sand and the dirt, they are walking through the dirt, through the dust and they don't have money to wash with soap."

### Perceived geographic variation in RTIs

Table 2 illustrates the results of a participatory voting exercise (N = 23 participants) where each participant was given 8 beans to distribute among the four zones according to the perceived geographic variation in occurrence of the condition in question. If a participant placed one or two beans on each of the four local terms for RTIs (vaginal discharge, inflamed ovaries, urinary tract infection, sexually-transmitted infections) and AIDS, the interpretation was that the participant did not perceive there to be a strong association between illness occurrence and the four different environments (rural highlands, marginal/recently settled areas of Las Pampas, modern/more established areas of Las Pampas and urban Lima), while placing all or most beans on one environment was interpreted as a strong association between illness occurrence and environment.

**Table 2:** Perceived relative abundance/frequency of reproductive tract infections in four different geographic areas, as assessed through a participatory voting exercise with 23 representatives of Las Pampas. Each participant was given 8 beans to distribute among the four areas according to the perceived geographic variation in occurrence of the condition in question. We interpreted an equal distribution of beans throughout the pictures (2 beans per picture) as no geographic association, whereas 8 beans on one picture was interpreted as high geographic association.

Geographic areas	Percent of beans assigned to each geographic area (Assessment performed separately for each illness term)					Allocation of beans across all five conditions
		
	Vaginal Discharge	Inflamed Ovaries	Urinary Tract Infection	AIDS	Sexually transmitted infections	
Rural-Highlands	9%	17%	13%	9%	10%	12%
Marginal-Pampas	67%	59%	70%	26%	39%	53%
Modern-Pampas	21%	18%	11%	23%	23%	19%
Urban Lima	3%	8%	6%	43%	28%	16%
Total Beans Assigned	184	184	159*	160*	145*	832

* Some respondents expressed no opinion regarding where certain conditions were most common, and therefore did not allocate all of their beans.

Table 2 shows that three of the conditions, vaginal discharge, inflamed ovaries and urinary tract infection were rated by respondents during the voting exercise as far more common in the marginal areas of Las Pampas. Sexually-transmitted infections were rated as slightly more common in the marginal areas of Las Pampas, while AIDS was rated as more common in urban Lima. To explain these results, it is necessary to consider once more the three most common categories of perceived causes for RTIs (Table 1): sexual contact with infected persons, personal hygiene and contaminated physical environment.

Sexual contact, according to respondents, is associated with AIDS, and is uncommonly associated with vaginal discharge, inflamed ovaries and urinary tract infection. Urban Lima was associated with unsafe sexual practices, especially commercial sex work, and that is the main reason participants rated urban Lima as the most common site for AIDS and the second most common site for the occurrence of sexually-transmitted infections. When asked why, women responded that the social environment of Lima was responsible. A higher prevalence of prostitution and more liberal women inside the city were seen as reasons for these diseases.

"Maybe he can have other partners because he is here in Lima, here the girls offer themselves for sex."

The adverse environment is thought to be a major contributing factor to vaginal discharge, inflamed ovaries and urinary tract infection, and the marginal areas of Las Pampas are thought to have the most adverse environment. Neither vaginal discharge nor "infection in the ovaries" was viewed as a problem in urban Lima:

"They don't have this in Lima because Lima is clean."

### Features of the social environment that favour alternative explanations

"As women, we are always suffering, when we have sex we have pain or we get an infection."

Ascribing of RTIs to alternative explanations such as deficient personal hygiene or to the adverse physical environment occurs in a setting where women have little or no control over sexual decision-making. Gender roles in Peruvian society are strongly dictated by traditional Latin American interpretations of what it means to be a woman or man. These social dictates give men control over sexual decisions and often leave women unable to alter disease risk. A respondent explained her failed attempt at using protection with her husband:

"He doesn't want me to use protection because he thinks that then I could have sex with another person"

Economic and social dependence on men strongly affected the willingness of women to address concerns related to sex. Machismo present in many Latin American countries is viewed as an important factor in this.

"...Machismo is generally the result of lack of female education.... Because men provide economic support, females feel submissive under the male domain...you always live dependent on men.... Men don't lose."

"El hombre es hombre" or "men are men" was a quote used by three of the women participating in this study.

"...men are men...sometimes he uses you sexually, then what happens--- sometimes we forget how men are."

Our respondents were quick to dismiss male responsibility in sexual relations but some acknowledged men's role in disease occurrence.

"Men are men, they use pleasure how they want and they don't use protection and this is how the disease comes."

## Discussion

In this exploratory study, we examined the influence of the physical and social environment on women's perceptions of RTIs and gynaecologic symptoms. These data are of programmatic significance because they highlight reasons why women deny or underestimate their personal RTI risk, and can contribute to improved approaches to communicating with women about RTI prevention. Knowledge of sexual contact as one way of transmitting RTIs was high, particularly for AIDS. However, the perceived personal risk of acquiring other RTIs through sexual contact was low.

The stigma associated with RTIs in women hinders treatment and discourages communication with sexual partners about such infections [22,23]. This stigma contributes to models of gynaecologic symptoms and infections that exclude sexual contact with infected persons as an explanation.

At the same time, other features of the physical and social environment may affect the quality of life on a daily basis and "pull" people toward external explanations for RTIs. Examples of such explanations reported in other studies include poor personal hygiene related to environmental contamination and lack of water, an adverse physical environment that contributes to exposure to dust, dirt, and cold, and excessive physical labour and other strenuous activities.

Women in this study associated gynaecologic symptoms and infections with aspects of their lives such as the physical environment, poverty, and male sexual behaviour that they are unable to alter. This situation may prevent women from using appropriate preventive measures or from seeking treatment. The ever-present dust, sand, and refuse that women encounter living in this community also lead them to feel that ill-health is inevitable. Participants were, nevertheless, correct in their perception that there is geographic variation in the occurrence of RTIs. For example, recent studies in Baltimore have demonstrated distinct clustering of gonorrhoea transmission [24]. However, the perception that rural areas are at lower risk and coastal urban areas are at higher risk for RTIs is partially contradicted by evidence from a study among a sample of women from the jungle, highland and coastal regions of Peru. The study found bacterial vaginosis was more common in Aymara-speaking women and vaginal trichomoniasis was associated with both highlands and coastal residence, and women from the coastal sample had a lowest mean number of lifetime partners than women in the jungle and highland areas [4].

## Conclusion

In this study, women described agents of contamination as microbes found in the dust and soil. This represents an area of common ground between local and 'cosmopolitan' or 'modern' medicine that may be targeted for prevention efforts. Both systems are concerned with preventing contact with illness causing agents, which both systems define as microbes. Since study participants seemed to understand the transmission of these microbes by sexual contact as well, this provides a good starting point for prevention efforts. Also, the perception that there is geographic variation in the occurrence of RTIs is correct, and could be the starting point for dialog on RTIs, although there are important differences between perceived variation and actual patterns of RTI prevalence as documented in recent prevalence surveys [4,5].

While further education about the causes, prevention and treatment of RTIs is warranted in this population, an argument can also be made for also investing resources in the physical infrastructure of peri-urban communities, including provision of potable water, wastewater disposal, refuse collection and improvements in local transport. Interventions that address RTI education and prevention alongside legitimate environmental concerns of the community are likely to be effective in a population where the environmental deterioration is closely linked to poor health standards as is the case in Las Pampas. The reproductive health agenda cannot be divorced from environmental health problems.

## Competing interests

There are no competing interests for this study. The study was funded in part by an ITREID training grant from the Fogarty Center, NIH (Bethesda, MD) and the RG-ER Anonymous Fund for Tropical Medicine Research, a private gift fund established by two American donors.

## Authors' contributions

LSH performed many of the interviews, trained and supervised other qualitative interviewers, coded and analyzed the data, and wrote the first draft of the paper. PJW provided technical input to LSH, carried out the literature review including Table 1, and edited the final draft of the paper. KP wrote the final draft of the paper and assisted with the literature review. RHG conceived of the study, participated in its design and coordination and helped to draft the manuscript. All authors read and approved the final manuscript

## Pre-publication history

The pre-publication history for this paper can be accessed here:


